# The association between sleep duration, respiratory symptoms, asthma, and COPD in adults

**DOI:** 10.3389/fmed.2023.1108663

**Published:** 2023-04-17

**Authors:** Zhishen Ruan, Dan Li, Xiaomeng Cheng, Minyan Jin, Ying liu, Zhanjun Qiu, Xianhai Chen

**Affiliations:** ^1^The First Clinical College, Shandong Chinese Medical University, Jinan, China; ^2^College of Traditional Chinese Medicine, Shandong Chinese Medical University, Jinan, China; ^3^Affiliated Hospital of Shandong University of Traditional Chinese Medicine, Jinan, China

**Keywords:** sleep duration, cough, wheezing, dyspnea, chronic obstructive pulmonary disease, asthma

## Abstract

**Introduction:**

The association between sleep duration and cough, wheezing, and dyspnea was unclear. This research aimed to test this relationship.

**Methods:**

Research data were obtained from people who participated in the National Health and Nutrition Examination Survey (NHANES) from 2005 to 2012. We used weighted logistic regression analysis and fitted curves to explore the association between sleep and respiratory symptoms. In addition, we investigated the association between sleep duration, chronic obstructive pulmonary disease (COPD), and asthma. The stratified analysis is used to analyze inflection points and specific populations.

**Results:**

The 14,742 subjects are weighted to reflect the 45,678,491 population across the United States. Weighted logistic regression and fitted curves show a U-shaped relationship between sleep duration and cough and dyspnea. This U-shaped relationship remained in people without COPD and asthma. The stratified analysis confirmed that sleep duration before 7.5 h was negatively associated with cough (HR 0.80, 95% CI 0.73–0.87) and dyspnea (HR 0.82, 95% CI 0.77–0.88). In contrast, it was positively associated with cough and (HR 1.30, 95% CI 1.14–1.48) dyspnea (HR 1.12, 95% CI 1.00–1.26) when sleep duration was >7.5 h. In addition, short sleep duration is associated with wheezing, asthma, and COPD.

**Conclusion:**

Both long and short sleep duration are associated with cough and dyspnea. And short sleep duration is also an independent risk factor for wheezing, asthma, and COPD. This finding provides new insights into the management of respiratory symptoms and diseases.

## Introduction

Coughing, wheezing, and dyspnea are common respiratory symptoms in adults. Two studies show that more than 50% of adults have at least one respiratory symptom (1, 2). Frequent respiratory symptoms bring attention to how these symptoms affect health. The research found that respiratory symptoms were associated with impaired quality of life even in the general population without chronic obstructive pulmonary disease (COPD) and asthma (3). In addition, in the general population, the presence of respiratory symptoms can increase the risk of all-cause mortality (4, 5).

Proper sleep duration is necessary for good health, and the American Sleep Foundation supports a daily sleep duration of 7–8 h for adults (6). However, since 1985, the proportion of adults with ≤6 h of sleep has gradually increased (7). Short sleep duration is considered a public health epidemic, associated with cardiovascular disease, obesity, and cancer (8). Moreover, long sleep duration is a growing concern and is a risk contributor to mortality and morbidity (9, 10).

Both long and short sleep duration seems to be factors affecting health, while the association between sleep duration and cough, wheezing, and dyspnea was unclear. Therefore, we plan to conduct studies to analyze the relationship between different sleep durations and coughing, wheezing and dyspnea. We also plan to analyze the relationship between sleep time, asthma, and COPD. The study population is from the 2005 to 2012 National Health and Nutrition Examination Survey (NHANES).

## Materials and methods

### Study population

National Health and Nutrition Examination Survey is a study based on the entire US population. Data collection included home screening, interviews, and physical examination (11). Each year, the NHANES staff selects a sample of 15 counties (about 5,000 people) from across the United States and calculates the sampling weights. With complex sampling weights, the population sampled reflects the overall US population. All information from the NHANES project is available on the official NHANES website (12). These data are de-identified and open to the public and therefore do not require the consent of the medical ethics committee.

Data for the study were obtained from participants who participated in the Sleep Duration Questionnaire from 2005 to 2012. The participants who performed the respiratory symptoms questionnaire were ≥40 years, so we only covered this subset of subjects. Supplementary Figure 1 illustrates the detailed inclusion and exclusion criteria.

### Sleep duration

The sleep duration is the answer to the question: “How much sleep {do you/does SP} usually get at night on weekdays or workdays?”. Sleep duration is classified as short sleep duration (<7 h), normal sleep duration (7–8 h), or long sleep duration (>8 h). In addition, we defined sleep disorders as an affirmative answer to the following question “Ever told doctor had trouble sleeping?”.

### Respiratory symptoms

Cough was classified as answering yes to the following questions. “Do you usually cough on most days for three consecutive months or more during the year?” to determine. Wheezing and dyspnea were defined as affirmative answers to the following questions. “In the past 12 months, have you had wheezing or whistling in your chest?” and “Have you had shortness of breath either when hurrying on the level or walking up a slight hill?”.

### Study covariates

We included demographic data to reduce potential bias, including gender, age, body mass index (BMI), race, smoking history, and education. Subjects receiving lung health questions were ≥40 years of age. The race is divided into White, Mexican American, Black, and Other races. Because of the small number of people with BMI <20, they were divided into three groups (<25, 25–30, and >30 kg/m^2^). Smoking status was categorized as never (smoking less than 100 cigarettes in a lifetime), previous (Smokes more than 100 cigarettes but has quit), and current.

Diabetes was defined as the presence of one of the following conditions (diagnosed by an internist, glycosylated hemoglobin ≥ 6.5%, fasting glucose ≥ 7.0 mmol/L, taking glucose-lowering medication, glucose tolerance test ≥ 11.1 mmol/L) (13). Hypertension was defined as the presence of one of the following conditions (diagnosed by a physician, taking antihypertensive medication, systolic blood pressure ≥ 140 mmHg, or diastolic blood pressure ≥ 90 mmHg) (14). The patient reports the presence of cardiovascular disease as the presence of any of the following (congestive heart failure, heart attack, coronary artery disease, stroke) (15). COPD is defined as a diagnosis of “chronic bronchitis or emphysema.” The presence of asthma was defined by the participant’s affirmative answer to the question, “Has a doctor or other health professional ever told you that you had asthma?” (16).

### Statistical analysis

Categorical data conforming to a normal distribution are described as numbers (percentages), and continuous variables are means ± standard deviations. Skewed data are represented by the median (25th–75th percentile). Kruskal Wallis and Chi-square (or Fisher’s exact) tests compare covariates.

Based on the sampling weights of the data, we used logistic regression and fitted curves to investigate the relationship between sleep duration and cough, wheeze, and dyspnea. We also investigated the relationship between sleep disorders and respiratory symptoms. Three models were adjusted to ensure the stability of the results. Furthermore, we also investigated the relationship between sleep duration, COPD, and asthma. Subgroup and stratification analyses were used to explore curve relationships and population classification.

The missing data for BMI was 5.3%, and we applied multiple interpolations to adjust the data. We also analyzed the data’s sensitivity before interpolating to reduce the error. The analysis was performed using R V4.1.3 and free statistical software (1.7).

## Results

### Baseline characteristics

A total of 14742 subjects were enrolled in our research (Supplementary Figure 1). After weighting, these participants reflect a population of 45,678,491 across the United States. Table 1 shows that 5,897 people had <7 h of sleep, 7,682 people had 7–8 h, and 1,163 people had >8 h of sleep. Comorbidities and respiratory symptoms were lower in the 7–8 h sleepers compared to the other two groups.

**TABLE 1:** Characteristics of participants, 2005–2012 NHANES (*n* = 14,742).

Variables	Sleep duration (h)			*P*
	**7–8 (*n* = 7,682)**	**<7 (*n* = 5,897)**	**>8 (*n* = 1,163)**	
Age, mean ± SD	60.4 ± 12.7	58.6 ± 12.1	66.1 ± 13.2	<0.001
Female, *n* (%)	3896 (50.7)	2959 (50.2)	641 (55.1)	0.008
Race/ethnicity, *n* (%)				<0.001
Non-Hispanic White	4,054 (52.8)	2,346 (39.8)	668 (57.4)	
Mexican American	1,152 (15.0)	824 (14.0)	148 (12.7)	
Non-Hispanic Black	1,297 (16.9)	1,723 (29.2)	225 (19.3)	
Other race	1,179 (15.3)	1,004 (17.0)	122 (10.5)	
BMI*, *n* (%)				<0.001
<25	1,975 (27.2)	1,346 (24.0)	313 (29.5)	
25–30	2,684 (37.0)	1,941 (34.6)	338 (31.9)	
>30	2,604 (35.9)	2,317 (41.3)	409 (38.6)	
Smoke, *n* (%)				<0.001
Never smoker	3,983 (51.8)	2,879 (48.8)	557 (47.9)	
Former smoker	2,446 (31.8)	1,694 (28.7)	388 (33.4)	
Current smoker	1,253 (16.3)	1,324 (22.5)	218 (18.7)	
Education, *n* (%)				<0.001
< High school diploma	1,127 (14.7)	878 (14.9)	237 (20.4)	
Completed high school	2,880 (37.5)	2,403 (40.7)	471 (40.5)	
≥College	3,675 (47.8)	2,616 (44.4)	455 (39.1)	
**Comorbidities, *n* (%)**				
Asthma	805 (10.5)	901 (15.3)	141 (12.1)	<0.001
COPD	530 (6.9)	509 (8.6)	93 (8.0)	<0.001
CVD	1,132 (14.7)	1,030 (17.5)	307 (26.4)	<0.001
Diabetes	1,747 (22.7)	1,559 (26.4)	349 (30.0)	<0.001
Hypertension	4,082 (53.1)	3,400 (57.7)	737 (63.4)	<0.001
**Respiratory symptoms, *n* (%)**				
Cough	693 (9.0)	756 (12.8)	163 (14.0)	<0.001
Wheezing	933 (12.1)	1,027 (17.4)	160 (13.8)	<0.001
Dyspnea	2,240 (29.2)	2,263 (38.4)	440 (37.8)	<0.001

*Missing value: 815/14741, 5.53%. BMI, body mass index; COPD, chronic obstructive pulmonary disease; CVD, cardiovascular disease.

### Association between sleep duration and respiratory symptoms

Table 2 shows the relationship between sleep duration (<7, 7–8, >8 h) and respiratory symptoms. Using 7–8 h as a reference, short and long sleep duration was associated with a 41 and 56% increase in cough, respectively. Less sleep is also associated with the occurrence of wheezing and dyspnea.

**TABLE 2:** Association of sleep duration (h) and respiratory symptoms.

	Model 1 OR (95% CI)	*P*	Model 2 OR (95% CI)	*P*	Model 3 OR (95% CI)	*P*
**Cough**						
**Sleep duration (h)**						
7–8	1(Ref)		1(Ref)		1(Ref)	
<7	1.60 (1.36–1.88)	<0.001	1.50 (1.26–1.79)	<0.001	1.44 (1.20–1.73)	<0.001
>8	1.84 (1.54–2.21)	<0.001	1.63 (1.35–1.97)	<0.001	1.54 (1.27–1.87)	<0.001
**Wheezing**						
**Sleep duration (h)**						
7–8	1(Ref)		1(Ref)		1(Ref)	
<7	1.41 (1.26–1.58)	<0.001	1.26 (1.11–1.42)	<0.001	1.21 (1.07-1.37)	0.003
>8	1.17 (0.92–1.50)	0.205	1.13 (0.86–1.49)	0.361	1.08 (0.82–1.41)	0.587
**Dyspnea**						
**Sleep duration (h)**						
7–8	1(Ref)		1(Ref)		1(Ref)	
<7	1.51 (1.41–1.63)	<0.001	1.46 (1.31–1.62)	<0.001	1.39 (1.25–1.55)	<0.001
>8	1.48 (1.30–1.68)	<0.001	1.28 (1.08–1.52)	0.006	1.17 (0.98–1.40)	0.076

Model 1 adjusted nothing, model 2 was adjusted for age, sex, race, BMI, smoking, and model 3 was adjusted for model 2 plus education, cardiovascular disease, diabetes, and hypertension.

**FIGURE 1:**
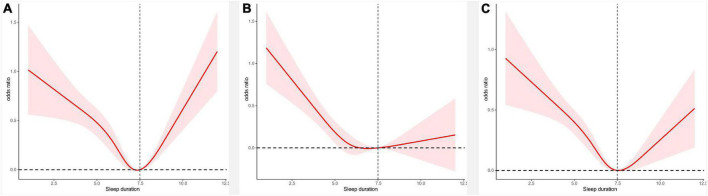
Panel **(A)** shows the curves of sleep duration and cough, panel **(B)** shows the curves of sleep duration and wheeze, and panel **(C)** shows the curves of sleep duration and dyspnea.

Figures 1A, C shows a U-shaped curve between sleep duration and cough and dyspnea. Based on this result, we performed a stratified analysis. The results in Table 3 support a U-shaped relationship between sleep duration and respiratory symptoms. Sleep duration was negatively correlated with cough (HR 0.80,95% CI 0.73–0.87) and dyspnea (HR 0.82,95% CI 0.77–0.88) before 7.5 h, whereas after >7.5 h, sleep duration was positively correlated with cough (HR 1.30,95% CI 1.14–1.48) and dyspnea (HR 1.12,95% CI 1.00–1.26). Supplementary Tables 1, 2 show the results before interpolation, in agreement with the main results.

**TABLE 3:** Stratified regression of sleep duration and respiratory symptoms.

Inflection point of sleep duration (7.5 h)	OR (95% CI)	*P*
**Cough**		
Sleep duration (h) <7.5	0.80 (0.73–0.87)	<0.001
Sleep duration (h) ≥7.5	1.30 (1.14–1.48)	<0.001
*P* for interaction		<0.001
**Wheezing**		
Sleep duration (h) <7.5	0.85 (0.79–0.92)	<0.001
Sleep duration (h) ≥7.5	1.07 (0.92–1.23)	0.378
*P* for interaction		0.005
**Dyspnea**		
Sleep duration (h) <7.5	0.82 (0.77–0.88)	<0.001
Sleep duration (h) ≥7.5	1.12 (1.00–1.26)	0.047
*P* for interaction		<0.001

Adjusted for age, sex, race, BMI, smoking, education, cardiovascular disease, diabetes, and hypertension.

Figure 1B shows the relationship between the curve of sleep duration and wheezing. Table 3 confirms a 13% reduction in the probability of wheezing occurring for each 1 h increase in sleep duration until 7.5 h. When >7.5 h, there was no statistical relationship between sleep duration and wheezing. In addition, Supplementary Table 3 shows that less sleep time is associated with COPD and asthma. Supplementary Table 4 shows that odds of coughing, wheezing, and dyspnea were higher in patients with sleep disorders. Supplementary Table 5 shows the results of the stratified analysis. As shown in Supplementary Figure 5, the relationship between sleep duration and respiratory symptoms was consistent with the primary results.

## Discussion

Our study found an association between sleep duration and cough, wheezing, and dyspnea. We found a u-shaped relationship between sleep duration and cough and dyspnea by fitting curves and stratified regression. It was negatively correlated with cough and dyspnea when sleep duration was less than 7.5 h, while this relationship was positively correlated when sleep duration was greater than 7.5 h. Shorter sleep duration increases the incidence of wheezing. In addition to respiratory symptoms, we found that less sleep duration was associated with asthma and COPD.

Cough, wheezing, and dyspnea are significant complaints and are often considered to be related to cardiopulmonary disease. These respiratory symptoms not only increase the risk of death due to lung disease, but are also associated with cardiovascular mortality and all-cause mortality (2, 17, 18). In addition, respiratory symptoms were an independent predictor of reduced lung function, COPD, and asthma (19–21).

Several studies have found a relationship between various sleep disorders and respiratory symptoms (22, 23). More respiratory symptoms were also present in people with habitual snoring (24). Bjornsdottir found that people with short sleep times reported more respiratory symptoms; not only that, they found that long sleep duration was associated with morning cough and dyspnea after activity (25). Unlike Bjornsdottir’s study, we did not identify a correlation between long sleep and dyspnea. This difference may be related to the definition of the length of sleep. Therefore, we were more interested in a linear relationship. We discovered a U-shaped association between sleep duration and cough and dyspnea, with the inflection point for optimal sleep duration being about 7.5 h.

The health hazards of sleep deprivation are well known, but the dangers of excessive sleep are easily overlooked (9). Study shows excessive sleep is an independent risk factor for metabolic syndrome, obesity, and depression (26–28). Long periods of sleep can also be potentially harmful (29). A meta-analysis showed that excessive sleep rather than less sleep duration was associated with increased systemic inflammatory biomarkers (CRP and IL-6) (30). Two studies show a U-shaped relationship between sleep and FeNO and lung function (31, 32). Several of the above studies help explain the curvilinear relationship between sleep, cough, and dyspnea.

In addition to respiratory symptoms, we found that short sleep duration was associated with asthma and COPD. Another study on NHANES also confirmed the association between less sleep duration and asthma (32). Among asthmatics, short sleep duration asthma attacks are more frequent, and long sleep duration people have more frequent activity limitations (33). Shorter sleep time is associated with the development of COPD and is an essential factor in the quality of life of COPD patients (34, 35). Adults with chronic airway obstructive disease are more likely to have sleep disorders (36). Given the association between sleep and respiratory disease, we performed additional subgroup analyses. In people without asthma and COPD (S4), sleep duration still has a U-shaped relationship with cough and dyspnea.

The current study has several advantages; professional data collection standards ensure accurate data. In addition, the data characteristics of the sample can reflect the characteristics of the overall U.S. population by weighting. Admittedly, our study has some limitations. First, the characteristics of the cross-sectional study led us not to determine the causal relationship between sleep duration and coughing, wheezing, and dyspnea. Second, self-reported sleep duration may not be as accurate as polysomnography. Third, we lacked factors regarding sleep duration and respiratory symptoms, such as sleep apnea, GERD, sleep quality, and insomnia. Forth, the diagnosis of COPD and asthma rely on patient self-report, which may create a potential bias. Finally, this was an analysis conducted in subjects greater than or equal to 40 years of age and the results do not generalize to those younger than 40 years of age.

## Conclusion

In conclusion, this research revealed a U relationship between sleep duration, cough, and dyspnea. And short sleep duration is also a risk factor for wheezing, asthma, and COPD. This finding provides new insights into the management of respiratory symptoms and diseases. In the future, more studies are needed to fully analyze all aspects of sleep (duration, quality) with respiratory symptoms (cough, wheezing, dyspnea) to understand more clearly about this relationship.

## Data availability statement

The original contributions presented in this study are included in the article/Supplementary material, further inquiries can be directed to the corresponding authors.

## Ethics statement

Study protocols for NHANES were approved by the NCHS Ethics Review Board (Protocol #2011–17, https://www.cdc.gov/nchs/nhanes/irba98.htm). All the participants signed the informed consent before participating in the study. The patients/participants provided their written informed consent to participate in this study.

## Author contributions

ZR and DL participated in the study design and edited the manuscript. ZR, DL, and XMC participated in the extraction and cleaning of data, and carried out the visualization analysis of the data. XMC, YL, and MJ participated in manuscript modification. ZQ and XHC participated in the research design and editor of the manuscript. All authors reviewed and approved the final manuscript.

## Supplementary material

Supplementary Figure 1 Flow chart of the study.
