# Supplementary material for: The association between sleep duration, respiratory symptoms, asthma, and COPD in adults
Source: Front Med (Lausanne). 2023 Apr 17;10:1108663. doi: 10.3389/fmed.2023.1108663 (PMC10150117; doi:10.3389/fmed.2023.1108663)
Supplement: Supplementary file 2 [file Data_Sheet_1.pdf]

Table S1 Association of Sleep duration (h) and respiratory symptoms (No multiple interpolation)

	**Model 1**<br>**OR (95% CI)**	***P***	**Model 2**<br>**OR (95% CI)**	***P***	**Model 3**<br>**OR (95% CI)**	***P***
**Cough**						
Sleep duration (h)						
7-8h	1(Ref)		1(Ref)		1(Ref)	
< 7h	1.60 (1.36-1.88)	<0.001	1.50 (1.26-1.79)	<0.001	1.46 (1.22-1.75)	<0.001
> 8h	1.84 (1.54-2.21)	<0.001	1.63 (1.35-1.97)	<0.001	1.58 (1.28-1.95)	<0.001
**Wheezing**						
Sleep duration (h)						
7-8h	1(Ref)		1(Ref)		1(Ref)	
< 7h	1.41 (1.26-1.58)	<0.001	1.26 (1.11-1.42)	<0.001	1.22 (1.04-1.32)	0.002
> 8h	1.17 (0.92-1.50)	0.205	1.13 (0.86-1.49)	0.361	1.14 (0.86-1.50)	0.348
**Dyspnea**						
Sleep duration (h)						
7-8h	1(Ref)		1(Ref)		1(Ref)	
< 7h	1.51 (1.41-1.63)	<0.001	1.46 (1.31-1.62)	<0.001	1.38 (1.23-1.54)	<0.001
> 8h	1.48 (1.30-1.68)	<0.001	1.28 (1.08-1.52)	0.006	1.20 (0.99-1.45)	0.061

Model 1 adjusted nothing, model 2 was adjusted for age, sex, race, BMI, smoking, and model 3 was adjusted for model 2 plus education, cardiovascular disease, diabetes, and hypertension.

Table S2 Stratified regression of sleep duration and respiratory symptoms (No multiple interpolation)

Inflection point of sleep duration (7.5h)	OR (95% CI)	*P*
**Cough**		
Sleep duration (h) < 7.5h	0.80 (0.73-0.87)	<0.001
Sleep duration (h) ≥ 7.5h	1.32 (1.15-1.52)	<0.001
P for interaction		<0.001
**Wheezing**		
Sleep duration (h) < 7.5h	0.85 (0.79-0.92)	<0.001
Sleep duration (h) ≥ 7.5h	1.11 (0.95-1.29)	0.193
P for interaction		0.002
**Dyspnea**		
Sleep duration (h) < 7.5h	0.83 (0.77-0.89)	<0.001
Sleep duration (h) ≥ 7.5h	1.14 (1.00-1.29)	0.046
P for interaction		<0.001

Adjusted for age, sex, race, BMI, smoking, education, cardiovascular disease, diabetes, and hypertension.

Table S3 Association of Sleep duration (h) and respiratory diseases (asthma and COPD)

	OR (95% CI)	*P*
**Asthma**		
Sleep duration (h)		
7-8h	1(Ref)	
< 7h	1.34 (1.16,1.56)	<0.001
> 8h	1.12 (0.84,1.50)	0.426
**Inflection point of sleep duration (7.5h)**		
Sleep duration (h) < 7.5h	0.78 (0.73,0.84)	<0.001
Sleep duration (h) ≥ 7.5h	1.06 (0.91,1.25)	0.445
P for interaction		<0.001
**COPD**		
Sleep duration (h)		
7-8h	1(Ref)	
< 7h	1.25 (1.06-1.46)	0.008
> 8h	1.01 (0.74-1.39)	0.935
**Inflection point of sleep duration (7.5h)**		
Sleep duration (h) < 7.5h	0.83 (0.77,0.91)	<0.001
Sleep duration (h) ≥ 7.5h	1.01 (0.85-1.21)	0.890
P for interaction		0.105

Adjusted for age, sex, race, BMI, smoking, education, cardiovascular disease, diabetes, and hypertension.

Table S4 Association of Sleep disorder and respiratory symptoms

	Model 1<br>OR (95% CI)	*P*	Model 2<br>OR (95% CI)	*P*	Model 3<br>OR (95% CI)	*P*
**Cough**						
**Sleep disorder**						
No	1(Ref)		1(Ref)		1(Ref)	
Yes	1.72 (1.51,1.97)	<0.001	1.57 (1.37,1.81)	<0.001	1.49 (1.30,1.72)	<0.001
**Wheezing**						
**Sleep disorder**						
No	1(Ref)		1(Ref)		1(Ref)	
Yes	2.61 (2.24,3.03)	<0.001	2.42 (2.04,2.87)	<0.001	2.29 (1.93,2.71)	0.002
**Dyspnea**						
**Sleep disorder**						
No	1(Ref)		1(Ref)		1(Ref)	
Yes	2.28 (2.03,2.55)	<0.001	2.10 (1.87,2.37)	<0.001	1.96 (1.73,2.22)	<0.001

Model 1 adjusted nothing, model 2 was adjusted for age, sex, race, BMI, smoking, and model 3 was adjusted for model 2 plus education, cardiovascular disease, diabetes, and hypertension.

Table S5 Subgroup analysis of sleep duration (hours) and respiratory symptoms

	Cough OR (95% CI)	*P*	Wheezing OR (95% CI)	*P*	Dyspnea OR (95% CI)	*P*
**COPD/asthma**						
Sleep duration (h) < 7.5h	0.82 (0.72-0.92)	0.001	0.91 (0.81-1.00)	0.043	0.87 (0.77-0.99)	0.037
Sleep duration (h) ≥ 7.5h	1.46 (1.13-1.89)	0.005	1.22 (0.95-1.56)	0.124	1.11 (0.82-1.49)	0.496
P for interaction	< 0.001		0.041		0.173	
**Non-COPD/asthma**						
Sleep duration (h) < 7.5h	0.84 (0.75,0.94)	0.003	0.92 (0.83,1.00)	0.046	0.84 (0.77-0.92)	<0.001
Sleep duration (h) ≥ 7.5h	1.26 (1.09,1.47)	0.002	1.00 (0.83,1.21)	0.995	1.14 (1.01-1.28)	0.033
P for interaction	< 0.001		0.336		< 0.001	
**Sleep disorder**						
Sleep duration (h) < 7.5h	0.77 (0.68-0.88)	<0.001	0.89 (0.82-0.97)	0.006	0.90 (0.82-1.00)	0.047
Sleep duration (h) ≥ 7.5h	1.26 (1.01-1.62)	0.024	1.12 (0.86-1.45)	0.387	1.10 (0.88-1.38)	0.396
P for interaction	< 0.001		0.072		0.040	
**Non-sleep disorder**						
Sleep duration (h) < 7.5h	0.88 (0.79-0.98)	0.021	0.95 (0.85-1.07)	0.407	0.83 (0.76-0.90)	<0.001
Sleep duration (h) ≥ 7.5h	1.32 (1.13-1.53)	<0.001	1.02 (0.88-1.19)	0.777	1.11 (0.99-1.24)	0.080
P for interaction	< 0.001		0.485		< 0.001	
**< 60 years**						
Sleep duration (h) < 7.5h	0.81 (0.72,0.90)	<0.001	0.84 (0.76,0.92)	<0.001	0.80 (0.73,0.89)	<0.001
Sleep duration (h) ≥ 7.5h	1.43 (1.12,1.83)	0.005	0.97 (0.79,1.19)	0.784	1.17 (0.96,1.43)	0.117
P for interaction	< 0.001		0.221		< 0.001	
**≥ 60 years**						
Sleep duration (h) < 7.5h	0.78 (0.71,0.85)	<0.001	0.87 (0.78,0.96)	0.008	0.84 (0.78,0.91)	<0.001
Sleep duration (h) ≥ 7.5h	1.25 (1.09,1.43)	0.002	1.15 (0.97,1.36)	0.105	1.08 (0.95,1.23)	0.217
P for interaction	< 0.001		0.018		0.001	

Adjusted for age, sex, race, BMI, smoking, education, cardiovascular disease, diabetes, and hypertension.
