# Supplementary figures and images for: The association between sleep duration, respiratory symptoms, asthma, and COPD in adults
Source: Front Med (Lausanne). 2023 Apr 17;10:1108663. doi: 10.3389/fmed.2023.1108663 (PMC10150117; doi:10.3389/fmed.2023.1108663)

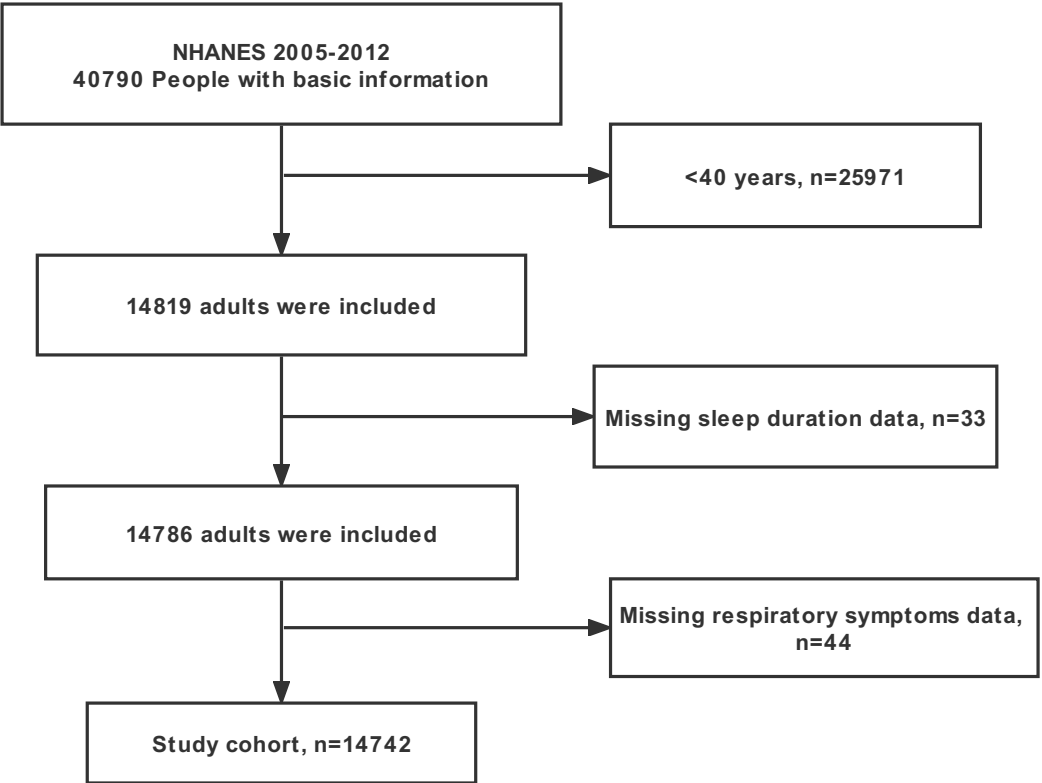

Supplement: Supplementary Figure 1 — Flow chart of the study. [file Image_1.pdf]
